# CD8 T cells protect adult naive mice from JEV-induced morbidity via lytic function

**DOI:** 10.1371/journal.pntd.0005329

**Published:** 2017-02-02

**Authors:** Nidhi Jain, Neelam Oswal, Amanpreet Singh Chawla, Tanvi Agrawal, Moanaro Biswas, Sudhanshu Vrati, Satyajit Rath, Anna George, Vineeta Bal, Guruprasad R. Medigeshi

**Affiliations:** 1 Immunobiology, National Institute of Immunology, New Delhi, India; 2 Vaccine and Infectious Disease Research Centre, Translational Health Science and Technology Institute, Faridabad, India; University of Liverpool, UNITED KINGDOM

## Abstract

Following Japanese encephalitis virus (JEV) infection neutralizing antibodies are shown to provide protection in a significant proportion of cases, but not all, suggesting additional components of immune system might also contribute to elicit protective immune response. Here we have characterized the role of T cells in offering protection in adult mice infected with JEV. Mice lacking α/β–T cells (TCRβ–null) are highly susceptible and die over 10–18 day period as compared to the wild-type (WT) mice which are resistant. This is associated with high viral load, higher mRNA levels of proinflammatory cytokines and breach in the blood-brain-barrier (BBB). Infected WT mice do not show a breach in BBB; however, in contrast to TCRβ-null, they show the presence of T cells in the brain. Using adoptive transfer of cells with specific genetic deficiencies we see that neither the presence of CD4 T cells nor cytokines such as IL-4, IL-10 or interferon-gamma have any significant role in offering protection from primary infection. In contrast, we show that CD8 T cell deficiency is more critical as absence of CD8 T cells alone increases mortality in mice infected with JEV. Further, transfer of T cells from beige mice with defects in granular lytic function into TCRβ-null mice shows poor protection implicating granule-mediated target cell lysis as an essential component for survival. In addition, for the first time we report that γ/δ-T cells also make significant contribution to confer protection from JEV infection. Our data show that effector CD8 T cells play a protective role during primary infection possibly by preventing the breach in BBB and neuronal damage.

## Author summary

Japanese encephalitis virus (JEV) commonly infects human beings in developing countries including those in Southeast Asia. While the majority of the infected people suffer from mild illness, a minority suffers from encephalitis which may lead to death. The virus is transmitted by mosquito bites and elimination of mosquitoes is not a practical answer to prevent the disease, therefore, prevention by vaccination is a desired goal. While various vaccines are clinically tried and some are marketed further improvement in vaccines is still possible. In a complex disease like JE many components of the immune system contribute to variable extent in protection. We show here that one subset of T cells called CD8 cells which are capable of killing infected cells are very critical for providing protection against JEV infection in mice. In the absence of T cells we also observed that virus reaches the brain early, unlike in the presence of T cells, and this possibly results in high virus load in the brain leading to worsening of the condition and death. Thus, our data help in identifying the role of CD8 T cells in protection from lethal JEV infection and the information may be useful for modifying and/or developing vaccine for prevention of JEV-mediated disease.

## Introduction

Japanese encephalitis virus (JEV) is a mosquito-borne flavivirus. JEV-mediated encephalitis is commonly found in South and Southeast Asia [1–4]. Although the ratio of clinical to subclinical illness is very low an estimated 67,900 cases are reported in Asia with 20–30% mortality [5]. In tropical countries like India around 1000 people die every year [6]. Owing to the enzootic life cycle of the virus, eradication at the vector level is almost impossible, thus the only feasible option for the prevention of disease is vaccination of the susceptible population in the endemic areas. While many vaccines are at various stages of development and some already marketed [7–9] recent work indicates that the present vaccines which work by triggering neutralizing antibody response may not be effective against newer genotypes [10]. Thus, whether vaccines which will generate effective T and B cell responses are likely to be better is not known. For addressing such questions animal models provide a clear opportunity.

Diverse clinical outcomes following infection in otherwise healthy humans imply that in addition to the dose of the virus and prior exposure to related viruses [11–14], the spectrum of morbidity can also be because of the extent and duration of inflammation associated with immune pathology [6]. Further, relatively low proportion of encephalitis cases amongst infected individuals suggests that in majority of the cases viral presence may be restricted to the extra neural tissues with immune responses and inflammation balancing each other without leading to major pathology. Thus, one of the key questions could indeed be identifying conditions that allow viral entry into the central nervous system (CNS) resulting in encephalitis.

Experimental models have demonstrated that B cells and antiviral antibodies play an important role in providing protection against JEV infection [15,16]. Antibodies can be detected in the sera as well as in the cerebrospinal fluid of symptomatic patients as early as day 5–7 post infection [17]. Based on vaccine studies there is evidence that neutralizing antibodies are protective in most instances. While high titers of neutralizing antibodies in humans correlate with protection, low levels are associated with morbidity and mortality [18], especially in the absence of neutralizing antibodies in the cerebrospinal fluid [19]. Protection offered by neutralizing antibodies against heterologous strains is less efficient [1,20]. In some mouse models CD4 T cells are documented to play a major role, however whether Th1-dominated or Th2-dominated responses offer better protection is still controversial [21,22]. While healthy humans exposed to JEV show a dominant CD8 response, patients show polyfunctional CD4 responses [23] providing a clear association for T cell contribution in protective response. However, there are still unanswered questions regarding the relative importance of CD8 T cells, NK cells and IFNγ [24–26]. Whether T cells expressing γ/δ receptor play any role is also not identified thus leaving the field open for further analysis of involvement of innate-like adaptive and adaptive immune components in protection from JEV infection.

We observe that adult mice lacking T cells expressing α/β receptor are highly susceptible to JEV infection after exposure to the virus and they also show a breach in BBB. Using adoptive transfer of cells from various gene-deficient mice we show that while CD8 T cells with functional granule-mediated cytolytic function are the major determinant of protective immune response, T cells bearing γ/δreceptor also provide some degree of protection from JEV infection.

## Methods

### Mice

Breeding pairs for all the strains were obtained from Jackson laboratories (Bar Harbor, USA) and mice were bred at the Small Animal Facility of National Institute of Immunology, New Delhi, India. Mice weighing 14–16 gm (4–6 wk old) of the following strains were used: C57BL/6 (B6)–wild-type (WT) mice and other congenic mutant strains—Rag1-null, TCRβ-null, TCRδ-null, TAP1-null, MHCII-null, Beige, IL-4-null, IL-10-null and IFNγ-null. All mice are from H-2b background.

### Ethics statement

Institutional Animal Ethics Committee of National Institute of Immunology (NII) reviewed and approved the proposal to carry out this work (Approval numbers: IAEC#216/09, IAEC#277/11 and IAEC#349/14). The animal care and use protocols used herein adhere to the relevant mandatory rules and regulations of the Government of India (http://cpcsea.nic.in/Content/54_1_ACTSANDRULES.aspx) as laid out in Indian law (The Prevention of Cruelty to Animals Act, 1960) and in the rules and regulations specified for it (The Breeding of and Experiments on Animals (Control and Supervision) Rules, 1998; amended, 2001; amended, 2006). NII registration number is 38/GO/Re Bi/SL/99/CPCSEA dated 15 December 2014.

### Virus propagation and titration

An Indian strain of JEV, P20778, was used throughout. Virus was propagated in infant WT B6 mice. On the third day following intracerebral (i.c.) inoculation of 10^5^ plaque forming units (PFUs) of virus mice showed symptoms of infection. A 10% suspension of infected mouse brain in 2% Minimum Essential Medium (MEM) with 10% fetal bovine serum was prepared, clarified of cell debris by low speed centrifugation, aliquots of the supernatant made and stored at -70°C. Viral titers were determined by plaque assay as described previously [27]. Viral burden in mouse brains was quantified by weighing the brain tissue, homogenizing in MEM and determining titers in the homogenate by plaque assay. Viral titers in whole blood were measured by serial dilution and plaque assay.

### Adoptive transfer of splenic cells

Spleen cells from various strains of sex-matched donor mice were harvested, RBCs removed by osmotic shock, washed and total spleen cells containing 5x10^6^ naïve T cells suspended in saline were given intravenously (i.v.) by the retro-orbital route to recipient TCRβ-null mice. Naïve T cells in the spleen were identified as CD3+CD44-CD62L+ by flowcytometry. For every adoptive transfer experiment, check staining was carried out on pooled spleen cells of the donor mice. Based on the proportion of total naïve T cells in spleen cell pool, as determined by flowcytometric staining, the absolute number of cells to be transferred to TCRβ-null recipients was determined. In different experiments, the range of spleen cells transferred varied between 6-7x10^7^. A representative staining profile and relative cell frequencies for major cell subsets in various mouse strains is shown in S1 Fig. The procedure for purification of total naïve T cells by electronic sorting is described below. 5x10^6^ sorted naïve T cells suspended in saline were similarly given i.v. Mice were infected 24 h later.

### Mouse infections

2.5x10^5^ PFUs of JEV resulted in ~ 90% mortality in TCRβ-null mice and ~20% mortality in WT B6 mice over 18–20 day observation period following i.v. infection. This dose was chosen for all the experiments. Mice were infected with 2.5x10^5^ PFUs of JEV i.v. and were analyzed for a period of 18 days for survival, weight loss and clinical score. Mice were weighed every third day and clinical score was measured every day post infection. In different experiments infected adult WT B6 mice showed between 75–100% survival, whereas infected TCRβ-null mice showed between 0–10% survival.

Clinical score was determined by the following parameters [28]:

0 = No restriction in movement, no pilo-erection, no body stiffening and no hind limb paralysis.
1 = No restriction in movement, no body stiffening, no hind limb paralysis but pilo-erection and slowness in movement.
2 = No restriction in movement, no body stiffening, no hind limb paralysis but pilo-erection, slowness in movement, slight hind limb extension, and stooping posture.
3 = Restriction in movement, pilo-erection, mild body stiffening, slight body jerks, slight hind limb extension but no hind limb paralysis.
4 = Restriction in movement, pilo-erection, body stiffening, hind limb paralysis, occasional tremor.
5 = Restriction in movement, pilo-erection, body stiffening, hind limb paralysis, tremor leading to death.

However, mice reaching stage 4 were euthanized to prevent further distress. All uninfected mice survived through the observation period.

### Evaluation of Blood Brain Barrier (BBB) permeability

BBB permeability changes after JEV infection were determined by measuring Evans blue (Qualigens, India) diffusion into the CNS as described previously [29]. Briefly, mice were injected intraperitoneally with 800 μl of 1% (w/v) solution of Evans blue dye. One hour later, mice were anesthetized and perfused with saline via intracardiac puncture. Brains were subsequently excised and photographed. Extent of blue color was then analyzed by ImageJ software.

### Isolation of leukocytes from brain

Leukocytes were isolated from the infected (day 12) and uninfected brains by Percoll gradient as described [30] with some modifications. Briefly, brains were harvested after perfusion of saline via intracardiac puncture. Single cell suspension was made using frosted slides and cell debris was removed by passing cell suspension through a 70 μm cell strainer. Using Percoll gradient cell pellet was collected, washed to remove Percoll and used further.

### Scoring of JEV-specific T cells in spleen cells

Infected WT B6 mice were euthanized on day 12 post-infection, single cell suspension made from spleens and RBCs lysed. Cells were stimulated with JEV (1 PFU/cell) for 12 hours, stained by fixable violet to identify live cells and fixed with 4% paraformaldehyde. They were stained for CD4, CD8, CD44 and CD69 as detailed below. Splenic cells from uninfected mice processed in parallel served as controls. Following published methodology [31], JEV-specific memory CD4 and CD8 T cells were identified as CD44highCD69+, since CD69 is an early activation marker for T cells.

### Flow cytometry for cellular analysis and sorting

Single cell suspension from spleens of WT B6, TCRβ-null, TCRδ-null, MHCII-null, TAP1-null and Beige mice were stained for CD3 (clone 145-2C11), CD4 (clone GK1.5), CD8 (clone 53–6.7), CD44 (clone IM7), CD62L (clone MEL-14), TCRδ (clone GL3), B220 (clone RA3-6B2), CD11b (clone M1/70) and NK1.1 (clone PK136) markers for phenotypic characterization. Cells were stained on ice for 1 h with antibody cocktails made out of various fluorophore-coupled antibodies to detect these surface markers, along with controls. Cells were washed and run on FACS-Verse (BD biosciences).

For staining leukocytes from brain suspensions fluorochrome labelled antibodies to detect CD45.2 (clone 104), CD3, CD4, CD8, TCRγ/δ, NK1.1, CD11b, Gr-1 (clone RB6-8C5), CD44 and CD69 (clone H1.2F3) were used. CD45.2 positive cells were identified as leukocytes. Further subsets were identified based on other markers.

Fluorochromes such as Fluorescein Isothiocyanate (FITC), Phycoerythrin (PE), PE coupled to Cy5.5 (PE-Cy5.5), Cy7 (PE-Cy7) or Texas-Red (PE-TxR), Allophycocyanin (APC), APC coupled to Cy7 (APC-Cy7), Pacific Blue, Alexa fluor and eFluor dyes or biotin conjugated to antibodies against various cell surface markers mentioned above were used in the study. All antibodies were purchased from BD Biosciences or eBioscience, USA. Representative staining profiles are shown in S1 Fig and S2 Fig.

For sorting total naïve T cells from B6 spleens, single cell suspensions were stained with CD3, CD25 (clone PC61), CD44 and CD62L. CD3+CD25-CD44-CD62L+ cells were sorted as naïve cells using FACSAria (BD Biosciences). Sorted cells were counted for viability and used.

### Detection of JEV-specific IgM and IgG by ELISA and neutralizing antibody assays

For determining JEV-specific antibodies in serum, ELISA plates were coated with 10^5^ U/ml of formalin inactivated virus. Serially diluted sera collected from mice before and after JE-infection were added. For IgM antibody detection optimal dilution of Fc-specific rabbit-anti-mouse IgM (1:20000) and for IgG detection Fc-specific rabbit-anti-mouse-IgG (1:5000) antibodies coupled to HRP were used (Both from Southern Biotech). In parallel, wells were coated with goat-anti-mouse Ig (Human ads-UNLB, cat# 1010–01, 1:500 dilution), normal mouse serum was used at multiple dilutions, and total IgM or IgG levels in non-immune mice were estimated using the same secondary reagents. These values were used to construct a standard curve and absolute amounts of JEV-antibodies in immune sera were calculated.

For detecting neutralizing antibodies, PS cells (10^5^cells/well) were grown in 24-well tissue culture plates for 24 hours. Serum samples were inactivated for 30 minutes at 56°C and serially diluted two-fold from 1:10 to 1:640 in virus medium (MEM containing penicillin/streptomycin antibiotics and 10^4^ pfu/ml virus). Virus-serum mix was added onto PS cells and incubated for 1 hour at 37°C. The mixture was removed from the cell monolayer and overlay medium was added. Plates were fixed and stained after two days as described earlier [27]. The neutralizing antibody titer (PRNT_50_) was defined as the reciprocal of the last serum dilution that showed 50% or more plaque reduction compared with the plaque counts in the control (mock-infected) serum well. PRNT_50_ titers ≥1:10 were considered positive.

### qRT PCR

Briefly, blood was collected before euthanasia from anaesthetized mice. Brains were weighed and homogenized in MEM. Total RNA was extracted using Trizol as per manufacturer’s instructions (Life technologies). For analysis of cytokine mRNAs (IL-1β, IL-6, IFNγ and TNFα) in brains of infected and control mice, cDNA was generated from RNA using SuperScript III First-Strand Synthesis System according to the manufacturer’s protocol (Life technologies). Relative gene expression was quantified by quantitative reverse transcription-PCR (qRT-PCR) with validated taqman primer-probe mixes (Assay IDs: Mm01336189_m1, Mm00446190_m1, Mm00168134_m1, Mm00443258_m1 respectively). Gene expressions were normalized to GAPDH copy numbers determined in parallel. Relative expression was calculated using comparative threshold cycle method and data is represented as fold up-regulation.

For detection of viral copy numbers one-step qRT-PCR was used as described previously [32], essentially using the same protocol for sample processing as described above and using GAPDH as a normalizing control. Viral titers were expressed as JEV genome equivalents/g tissue. Detection limit was 10^2^ copies/g tissue.

### Detection of cytokines in brain homogenate

Frozen brains were homogenized in cold lysis buffer and centrifuged at high speed to get supernatants following a published protocol [33]. These supernatants were used for protein and cytokine estimation. IL-1β, IL-6 and TNFα were detected using commercially available reagents (Biolegend) and recommended protocols. Total protein was estimated by micro BCA assay using a ready kit (Thermo Scientific). Cytokine values were normalized to protein values.

### Statistical analysis

Data from multiple experiments were pooled to calculate statistical significance using Mann Whitney U test. For survival analysis Kaplan Meier curves were plotted and statistical significance was calculated by log rank test using Graphpad Prism. p values of ≤ 0.05 were considered significant.

## Results

### Functional T cells are necessary to provide protection against primary JEV infection

**Fig 1:**
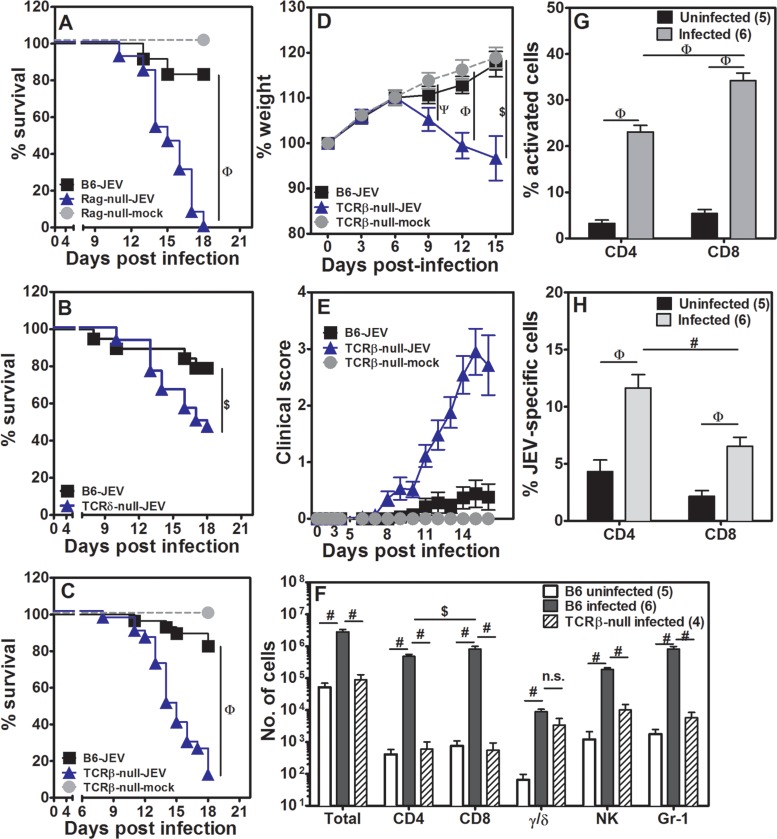
T cells play a significant role in protection from JEV infection. [A] Survival kinetics of JEV infected WT B6 and Rag1-null mice over time. n = 10–13 mice. [B] Survival kinetics of JEV infected WT B6 and TCRδ-null mice over time. n = 15–20 mice. [C] Survival kinetics of JEV infected WT B6 and TCRβ-null mice over time. n = 12–18 mice. [D] Relative weight loss of JEV infected TCRβ-null mice as compared to uninfected TCRβ-null (mock) and infected WT B6 mice (mean ± SE, n > 10). [E] Clinical score for JEV infected WT B6 and TCRβ-null mice along with uninfected TCRβ-null (mock) mice (mean ± SE, n > 10). [F] Distribution of leukocyte subsets per brain in uninfected WT B6, infected WT B6 and infected TCRβ-null mice (mean ± SE, n as shown). [G] Frequencies of CD44highCD69+ activated cells in brains of infected WT B6 mice (mean ± SE, n as shown). [H] Frequencies of CD44highCD69+ JEV-specific cells in spleens of infected WT B6 mice (mean ± SE, n as shown). $ = p<0.05, ψ = p<0.01, # = p<0.005, φ = p<0.001.

Both innate and adaptive immune components are reported to contribute to protection from JEV infection. We used Rag1-null mice to examine the role of adaptive immune response in protection against JEV infection in adult mice. Wild-type B6 mice and Rag1-null mice were given 2.5x10^5^ PFU of virus i.v. and observed over 18 days for weight-loss, clinical score and mortality. Mock infected Rag1-null mice survived throughout the observation period but none of the infected Rag1-null mice survived (Fig 1A). Mock infected WT B6 mice showed no mortality in any of the experiments. These data indicate that the presence of adaptive immune components is essential for the survival of mice over a sub-acute course of infection.

We next examined the role of γ/δ TCR bearing T cells in JEV infection. Consistently only ~50% of the infected TCRδ-null mice stayed alive by day 18 as compared to WT B6 mice (Fig 1B). Uninfected TCRδ-null mice showed no mortality. JEV infection of mice deficient in α/β TCR bearing T cells was tested next. Unlike TCRδ-null mice, only ~10% of infected TCRβ-null mice survived (Fig 1C) whereas all uninfected TCRβ-null mice were alive at the end of the observation period. We further characterized these mice for weight-loss and clinical score. While infected B6 mice and uninfected TCRβ-null mice showed weight-gain over the observation period infected TCRβ-null mice started showing weight-loss after day 6. By 15 days those TCRβ-null mice which had survived showed significant weight loss (Fig 1D). The condition of infected TCRβ-null mice started deteriorating from day 6 post-infection as is apparent from the clinical scores (Fig 1E) showing a correlation with weight-loss. As expected, there was no change in clinical scores of uninfected TCRβ-null mice and only a marginal, statistically insignificant, increase in infected B6 mice.

Since majority of the WT B6 mice showed no deterioration post-infection we examined the brains of these mice to see a direct role, if any, of T cells on day 12 post-infection. A representative staining strategy for identifying total leukocytes as CD45+ve cells (S2A Fig) and further subsets showed presence of NK cells (NK1.1+ve) and phagocytic cells (Gr-1+ve) (S2B Fig). All CD11b-expressing cells of myeloid origin were also Gr-1+ve at this stage of infection. Further, T cell subsets were identified as CD3+, CD4+, CD8+ and γ/δ+ve cells (S2C–S2E Fig). Specifically, at 12 days post-infection in the WT B6 mouse, the brain showed substantial infiltration with leukocytes with a prominent CD8 T cell component (Fig 1F). Conversely, at day 12 post-infection, the TCRβ-null mouse showed almost no leukocyte infiltration in the brain, associated with early morbidity. The numbers in infected TCRβ-null mice were nearly 30 fold lower than in infected WT B6 mice, but comparable to uninfected B6 mice (Fig 1F) despite showing high clinical score at this point of time. Numbers of infiltrating NK cells and phagocytic cells were also low in infected TCRβ-null mice as compared to infected WT B6 mice (Fig 1F). Numbers of γ/δ T cells did not differ between the two groups of infected mice (Fig 1F). Further analysis of the CD4 and CD8 T cells showed them as CD44hi and a subset showing CD44highCD69+ phenotype indicative of antigen-experienced cells activated in situ (S2F Fig). Data from multiple infected WT B6 mouse brains showed that a higher frequency of CD8 T cells were antigen-experienced as compared to CD4 cells (Fig 1G). These data indicate that CD8 T cells might be critical in mediation of protection against JEV morbidity in the mouse.

Since we had infected mice intravenously peripheral lymphoid organs, especially spleen, was expected to be the primary site of viral residence and replication thereby triggering immune response. In preliminary experiments we could detect low levels of virus in spleens and brains of infected WT B6 and TCRβ-null mice day 2 and 4 post-infection (S3 Fig). We also characterized spleen cells from JEV-infected WT B6 mice, by re-stimulating them with JEV and identifying antigen-specific CD4 and CD8 T cells by induction of CD69 expression on them. Both CD4 and CD8 T cells specific for JEV could be detected as seen in a representative plot (S2G and S2H Fig). Compiled data show that cells from infected WT B6 mice have higher frequency of both CD4 and CD8 memory cells as compared to uninfected control spleen cells. Interestingly the frequency of JEV-specific memory CD4 cells was much higher than that observed for CD8 T cells (Fig 1H), unlike in the brain, suggesting that in spleen both CD4 and CD8 T cells get primed efficiently.

### Both TCRβ-null and TCRδ-null mice show neutralizing antibody response

**Fig 2:**
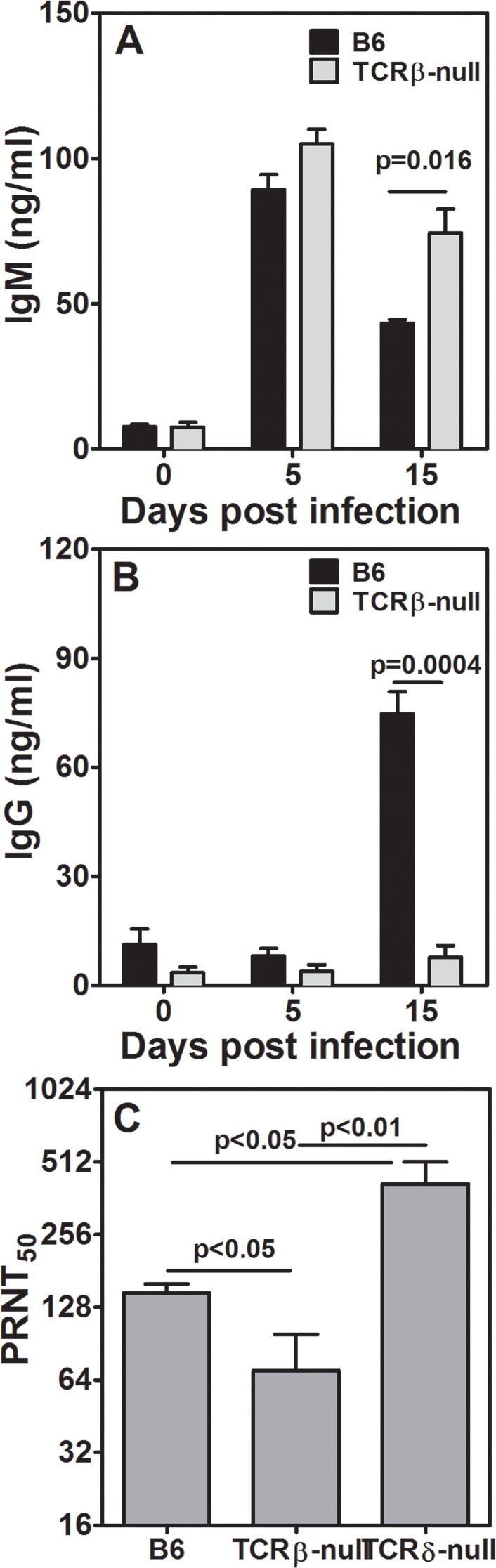
JEV-specific IgM, IgG and neutralizing antibody levels. [A] JEV-specific IgM levels in WT B6 and TCRβ-null mice prior to (day 0) and post-infection on indicated days. n = 6 mice. [B] JEV-specific IgG levels in WT B6 and TCRβ-null mice prior to (day 0) and post-infection on indicated days. n = 6 mice. [C] Virus neutralization titers (PRNT50) in infected WT B6, TCRβ-null and TCRδ-null shown as reciprocal log 2 values. n = 5 mice per group.

There are reports which document a variable role for antibodies, especially neutralizing antibodies, in protection against JEV infection in mice and humans [8,10,22,34]. We estimated JEV-specific IgM and IgG antibodies in WT B6 and TCRβ-null mice. IgM antibody levels on day 5 were higher than those seen prior to infection (day 0) in both WT B6 and TCRβ-null mice (Fig 2A). While IgM antibody levels decreased from day 5 to day 15 post-infection in both sets of mice, the drop in IgM antibody levels was much more pronounced in B6 mice as compared to TCRβ-null mice (Fig 2A). There was no appreciable increase in IgG antibody levels by day 5 in either strain of mice. Levels increased from day 5 to day 15 in B6 mice whereas in the absence of CD4 T cells TCRβ-null mice continued to show background IgG levels as expected (Fig 2B). We next examined the neutralizing potential of these antibodies. Serum from infected WT B6 mouse showed a significant inhibition in the number of plaques (S4A Fig, right panel), whereas serum from infected TCRβ-null mouse showed relatively lesser effect on plaque numbers (S2C Fig, right panel). Pooled data to show average PRNT_50_ titers confirm that on day 12 post-infection both WT B6 and TCRδ-null mice sera show much higher antibody response as compared to that seen from TCRβ-null mice (Fig 2C). Thus, TCRβ-null mice show clear presence of neutralizing antibodies, however, in the absence of α/β TCR bearing T cells these antibodies are not able to provide adequate protection from JEV. To further elucidate the role of T cells in JEV infection we focused on TCRβ-null mice.

### TCRβ-null mice show increased viral load and breach in BBB as compared to the WT B6 mice

**Fig 3:**
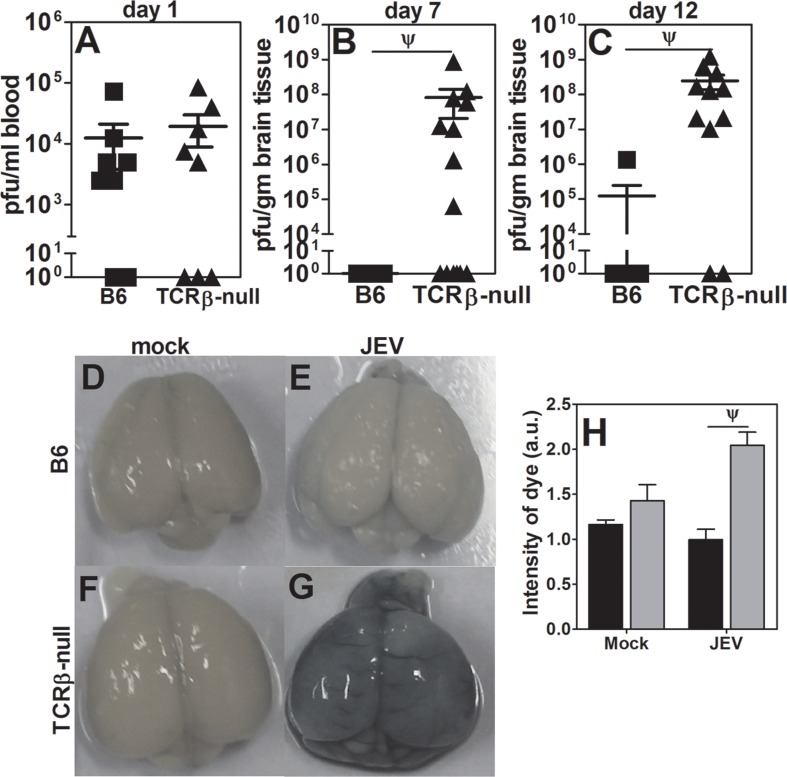
JEV burden in the blood and brains of B6 and TCRβ-/- mice. [A] JEV levels in blood at day 1 post infection (mean ± SE, n > 8). JEV levels in brain tissue at day 7 [B] and day 12 [C] post infection respectively (mean ± SE, n ≥ 8). [D-G] Representative images of brains after injection of Evans blue in WT B6 and TCRβ-null mice. [H] Pooled data to show relative levels of Evans blue in B6 (black bars) and TCRβ-null (gray bars) in mock and JEV-infected mice (mean ± SE, n > 5 mice). ψ = p<0.01.

To further understand the role of α/β TCR-bearing T cells we examined the kinetics of viral load in blood, spleen, liver, intestine, kidney and brain of WT B6 and TCRβ-null mice. On day 1 post-infection viral load measured as PFU/ml of blood was comparable in B6 and TCRβ-null mice (Fig 3A). Virus was not detectable in blood or brain on day 3–4 post-infection by this assay. However, in preliminary experiments, on day 2 and 4 post-infection the virus could be detected in the brain and spleen from infected TCRβ-null mice by real time PCR at low levels (~10^3^ genome equivalents/gm tissue, with 10^2^ as the detection limit) (S3 Fig). Similarly low copy numbers of virus were also detectable in infected B6 mice in brain, kidney and spleen on day 4 (S3 Fig). Virus could be detected in high titers in the brains of infected TCRβ-null mice by day 7. At this time point WT B6 mice had no detectable virus in the brain (Fig 3B). By day 12 there was marginal increase in viral titers in brains of TCRβ-null mice and only one out of 11 infected WT B6 mice showed detectable virus (Fig 3C). These data suggest that high viral titers develop in the absence of α/β TCR bearing T cells in the brain leading to death. Our findings that infected WT B6 mice very rarely show high viral titers in brain is similar to the observations reported earlier [15]. Since very high titers of the virus were detected in brains of infected TCRβ-null mice we examined the status of BBB in these mice. Infected TCRβ-null mice which showed neurological symptoms on day 7 post-infection showed breach in BBB, but those not showing neurological symptoms did not. By day 12 post-infection most of the TCRβ-null mice showed neurological symptoms. Representative images of the brain from mock- and JEV-infected brains from WT B6 mice do not show any breach in BBB at this time point (Fig 3D and 3E), whereas similar images from brains of uninfected (Fig 3F) and infected (Fig 3G) TCRβ-null mice showed a clear difference with brain from infected TCRβ-null mouse staining with Evans blue indicative of a breach in BBB. Pooled data from multiple mice in each category showed that the dye extravasation into the brain was significantly higher in infected TCRβ-null mice as compared to other groups (Fig 3H). TCRβ-null mice show increased levels of pro-inflammatory cytokines in brain as compared to WT B6 mice.

Depending upon the route of inoculation as well as the strain of the virus, peripheral tissues do or do not show presence of the virus [35,36]. In our case, absence of α/β TCR bearing T cells appeared to predispose mice to increased viral load in the brain with passage of time. We next examined possible causes of the breach in the BBB.

**Fig 4:**
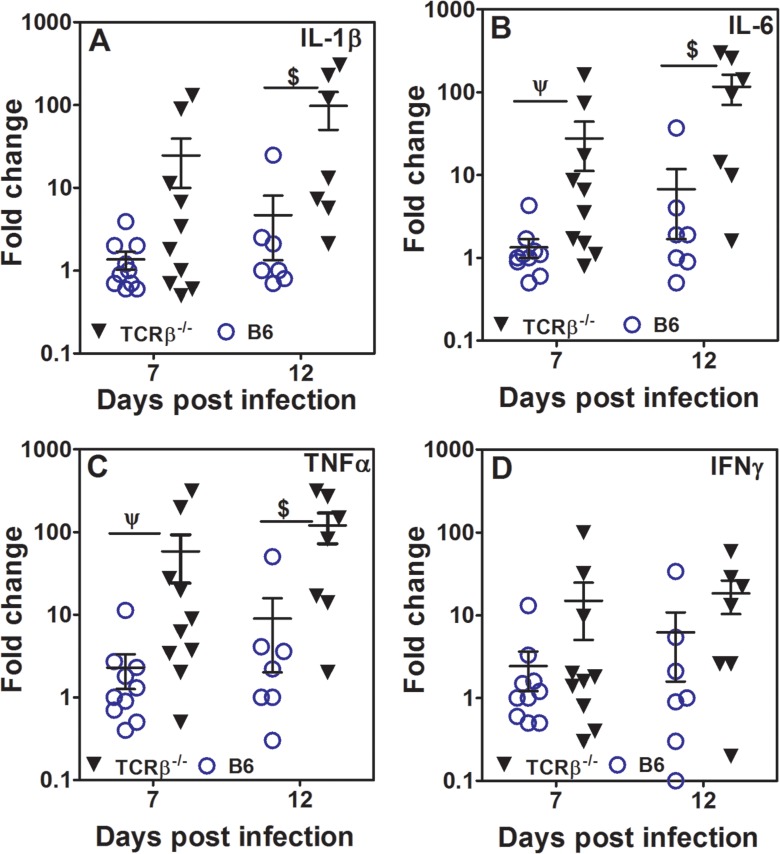
Cytokine mRNA levels in the brains of infected B6 and TCRβ-/- mice. Relative mRNA levels of IL-1β [A], IL-6 [B], TNFα [C] and IFNγ [D] normalized to GAPDH in the brain homogenates of B6 and TCRβ-null mice infected with JEV on day 7 and 12 post-infection (mean ± SE, n > 7). $ = p<0.05, ψ = p<0.01.

Earlier studies in JEV-infected patients have shown that the levels of IL-6, IL-8, TNFα and IFNα were significantly higher in the CSF of the non-survivors than in survivors [1]. In mice infected with JEV, a progressive decline in the levels of IL-4 and IL-10 has been reported [37]. We examined cytokine mRNA levels in WT B6 and TCRβ-null mice infected with JEV on day 7 and 12 post-infection. Brains from TCRβ-null mice showed significantly higher levels of pro-inflammatory cytokine mRNAs, i.e., IL-1β, IL-6 and TNFα as compared to that of B6 mice (Fig 4A–4C). By day 12 mRNA levels of these cytokines in B6 mouse brains were not much different except one mouse showing relatively high values as compared to day 7. This mouse did show higher clinical score as compared to the other infected B6 mice at this time point. Although TCRβ-null mice showed comparatively higher levels of IFNγ mRNA in the brain, the levels were quite variable and not significantly different as compared to B6 mice (Fig 4D). IL-4 and IL-10 mRNA levels were comparable at these time points in the brains of infected TCRβ-null or B6 mice. The respective values for IL-4 in infected B6 and TCRβ-null mice were 0.51 ± 0.02 and 0.50 ± 0.01. For IL-10 the values in infected B6 and TCRβ-null were 5.44 ± 3.9 and 7.30 ± 2.81 respectively. We also looked for cytokines in the brain by ELISA on day 12 post-infection. Only brain homogenates from infected TCRβ-null mice showed detectable TNFα and IL-6 levels, however, IL-1β was not detectable. Cytokine amounts (pg/ml) normalized to brain homogenate protein (mg/ml) are: TNFα = 10.0 +/- 0.9 pg/mg protein, IL-6 = 134.2 +/- 26.2 pg/mg protein (n = 3) in infected TCRβ-null brain homogenate.

### Transfer of naïve T cells bearing α/β TCR provide protection from JEV infection to TCRβ-null mice

**Fig 5:**
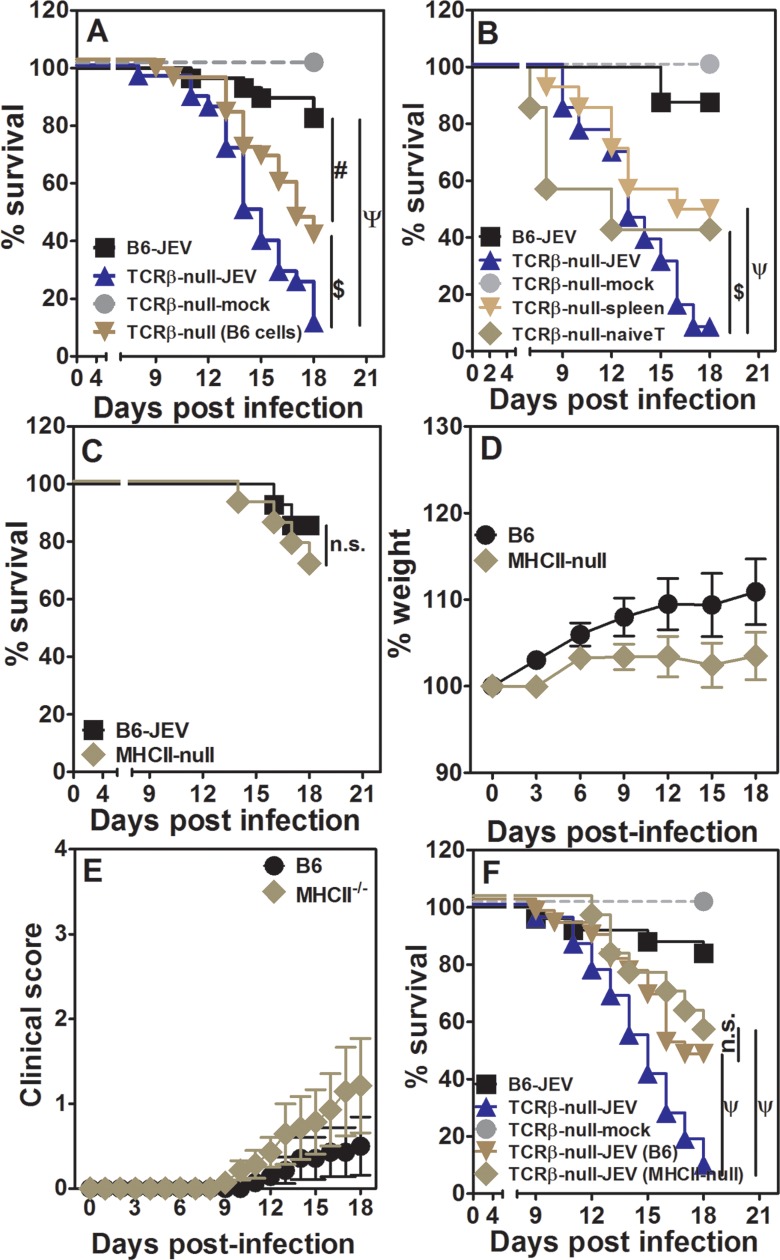
Presence of T cells is essential for protection from primary JEV infection, but CD4 T cells appear less critical. [A] Survival kinetics of mock and JEV infected TCRβ-null with or without transfer of naïve T cells from WT B6 mice as indicated (n > 8). [B] Survival kinetics of mock and JEV infected TCRβ-null mice with purified naïve T cell or total spleen cell transfers from WT B6 mice (n = 7 for sorted naïve T cell transfer, for other groups n > 8). [C] Survival kinetics following JEV infection in WT B6 and MHCII-null mice over time (n = 14). [D] Relative weight loss of JEV infected MHCII-null mice as compared to infected WT B6 mice (mean ± SE, n = 14). [E] Clinical score for JEV infected WT B6 and MHCII-null mice (mean ± SE, n = 14). [F] Survival kinetics of mock and JEV infected TCRβ-null mice with or without transfer of naïve T cells from MHCII-null or WT B6 mice (n > 8). $ = p<0.05, ψ = p<0.01, # = p<0.005, n.s. = not significant.

Since TCRβ-null mice lack only α/β TCR bearing T cells, we adoptively transferred splenic cells containing naïve T cells to test whether they would provide protection. Based on preliminary standardization experiments it was observed that a transfer of spleen cells containing 5x10^6^ naïve T cells from WT B6 mice provided ~50% survival rate (Fig 5A). Since our interest was to look for enhancement of, or decrease in, protection provided by naïve T cell transfers we used this cell number for future experiments. While most TCRβ-null mice which had not received spleen cells containing naïve T cells before infection (TCRβ-null-JEV) died by day 18, only ~50% mice receiving them succumbed to infection (Fig 5A). We also transferred 5x10^6^ electronically purified total naïve T cells from WT B6 mice to TCRβ-null mice and infected them with JEV. By day 18 post-infection only ~40% of the recipients of sorted naïve T cells survived whereas mice which received WT B6 spleen cells containing 5x10^6^ total naïve T cells showed marginally higher survival. However, there was no difference in the survival frequencies in these two groups (Fig 5B). These data unambiguously showed a dominant role for α/β TCR bearing T cells as contributors to the immune response and protection.

### In the absence of CD4+ T cells a high proportion of JEV-infected mice survive primary infection

The next question was to identify relative importance of subsets of α/β TCR bearing cells, namely CD4 and CD8. Previous studies have shown that adoptive transfer of JEV specific immune CD4 T cells to healthy mice protects the recipients from subsequent JEV challenge [15,21]. To dissect the role of CD4 T cells in providing protection against JEV infection in adult mice with mature immune system, MHC class II-deficient (MHCII-null) mice which lack mature peripheral CD4 cells were tested. There was no significant difference in the survival frequency of infected MHCII-null mice and WT B6 mice (Fig 5C). While infected MHCII-null mice did not gain as much weight as WT B6 mice did (Fig 5D), the difference in weights was not significant. Infected MHCII-null mice also showed somewhat higher clinical score as compared to WT B6 mice (Fig 5E) but that too was not significantly different. In contrast to infected TCRβ-null mice infected MHCII-null mice did not show any breach in BBB (Relative values for dye-extravasation in infected vs uninfected MHCII-nulls 1.07 +/- 0.08 vs. 1.00 +/- 0.03 from 4–6 mice per group). We further confirmed these data by adoptively transferring spleen cells containing ~5x10^6^ naïve T cells from MHCII-null mice to TCRβ-null mice and infecting the recipients. TCRβ-null mice which received spleen cells containing a mixture of CD4+ and CD8+ naïve cells from WT B6 mice served as a control for this series of experiments. TCRβ-null mice which received spleen cells containing majority of naïve CD8 T cells from MHCII-null mice post-infection showed marginally better survival than those mice receiving cells from WT B6 mice, however this difference was not statistically significant (Fig 5F). As expected TCRβ-null mice which received no cell transfer showed very high mortality post infection and infected WT B6 mice were resistant. These groups served as positive and negative controls. Together, these data suggest that CD4 T cells may not contribute significantly to offer protection from primary JEV infection.

### T cells deficient in secreting interferon-gamma (IFNγ), IL-4 or IL-10 do not contribute significantly to the outcome of JEV infection

**Fig 6:**
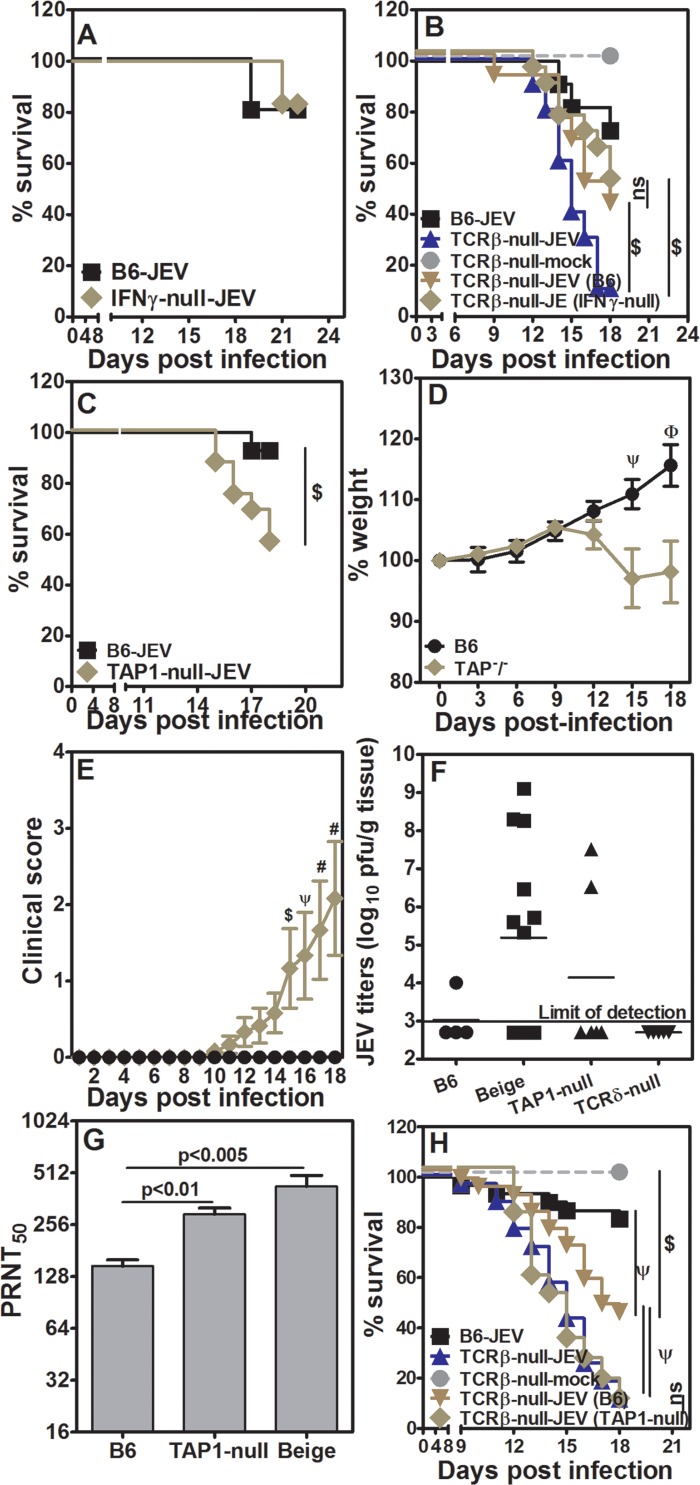
Absence of CD8 cells, but not IFNγ, influences the outcome of JEV infection. [A] Survival kinetics following JEV infection in WT B6 and IFNγ-null mice over time (n > 8). [B] Survival kinetics of mock or JEV infected TCRβ-null mice with or without transfer of naïve T cells from IFNγ-null or WT B6 mice (n > 8). [C] Survival kinetics following JEV infection in WT B6 and TAP1-null mice over time (n = 14). [D] Relative weight loss of JEV infected TAP1-null mice as compared to infected WT B6 mice (mean ± SE, n = 14). [E] Clinical score for JEV infected WT B6 and TAP1-null mice (mean ± SE, n = 14). [F] Titers of JEV in brains of infected WT B6, beige, TAP1-null and TCRδ-null mice day 12 post-infection. Each symbol represents data from individual mice. [G] Virus neutralization titers from WT B6, TAP1-null and beige mice day 12 post-infection (mean ± SE, n = 5). [H] Survival kinetics of mock or JEV infected TCRβ-null mice with or without transfer of naïve T cells from TAP1-null or WT B6 mice (n > 8). $ = p<0.05, ψ = p<0.01, # = p<0.005, φ = p<0.001, ns = not significant.

IFNγ is secreted by CD4+ and CD8+ T cells bearing α/β and γ/δ TCRs and NK cells. We first examined the role of IFNγ in anti-JEV immune response. WT B6 and IFNγ-null mice infected with JEV showed about 80% survival (Fig 6A). We next transferred splenic cells containing 5x10^6^ naïve T cells from IFNγnull mice to TCRβ-null mice with WT B6 spleen cell-transferred mice as controls. This permitted us to determine the relative role of IFNγ produced by endogenous γ/δ T cells and NK cells from TCRβ-null mice, in contrast to absence of IFNγ production by α/β TCR bearing transferred cells, in response to JEV infection. To our surprise, TCRβ null mice which received spleen cells from IFNγ-null mice containing 5x10^6^ total naïve T cells showed about 50% survival similar to those receiving WT B6 T cells (Fig 6B). These data suggest that IFNγ production from α/β TCR bearing CD4+ and CD8+ T cells may not be necessary for protection from JEV infection.

West Nile virus (WNV) infection results in increased levels of IL-10 and IL-10-null mice are more resistant to WNV infection [38]. In contrast, JEV infection in infant mice by intracranial route results in progressive decrease in IL-10 levels [39]. Hence we examined the role of IL-10 by infecting IL-10-null mice with JEV. IL-10-null mice were as susceptible to JEV infection as WT B6 mice (S5A Fig). When TCRβ-null mice received spleen cells equivalent of 5x10^6^ naïve T cells deficient in secreting IL-10 from IL-10-null mice, recipient TCRβ-null mice were marginally more resistant to death as compared to TCRβ-null mice receiving WT B6 cells (S5B Fig), however, there was no statistically significant difference between these two groups. These data indicate that IL-10 may not play any significant role in JEV-associated mortality in mice.

IL-4 secreted by immune CD4 T cells is associated with protection in JEV infection [22]. Similar to IL-10, IL-4 is also produced by T and non-T cells in the body. Hence we examined the consequences of JEV infection in IL-4-null mice. IL-4-null mice were as susceptible to infection as WT B6 mice (S5C Fig). In adoptive transfer experiment, TCRβ-null recipients given spleen cells containing 5x10^6^ total naïve T cells from IL-4-null mice showed marginally higher susceptibility to death as compared to those which received B6 cells (S5D Fig), this difference was also not statistically significant. These data suggest that IL-4 also does not make any difference to the outcome in terms of mortality.

### CD8 T cells contribute significantly to protection from JEV infection

Despite it being a viral infection, role of CD8 T cells in protection from JEV mediated disease is not clear. While adoptive transfer of immune CD8 cells from adult mice was not effective in offering protection in 14-day old mouse model [21], role of CD8 T cells was described as ‘subsidiary’ to antibodies in another report [15]. As shown above, our data showed a preponderance of CD8 T cells in infected WT B6 mouse brains (Fig 1F) but no clinical symptoms. On this backdrop we investigated the role of CD8 T cells in our adult mouse model.

TAP1-null mice have very small number of CD8 T cells in the periphery (S1E Fig) and hence we used these mice to evaluate the role of CD8 T cells in JEV infection. As compared to WT B6 mice TAP1-null mice showed poor survival (Fig 6C) clearly indicating that CD8 T cells are likely to have a role in protection against primary JEV infection associated mortality. Over the course of infection TAP1-null mice showed significantly more weight loss as compared to WT B6 mice (Fig 6D). Morbidity in infected TAP1-null mice became apparent from day 10–12 post-infection with significantly higher clinical scores (Fig 6E), unlike in TCRβ-null mice in whom clinical score started going up earlier (Fig 1E). We also observed that on day 12 post-infection about 30% of infected TAP1-null show presence of virus in the brain (Fig 6F). On day 12 days post-infection leukocytes from the brains of TAP1-null mice were quantified with uninfected mouse brains as controls. Unlike infected WT B6 mouse brains, there was only marginal but significant increase in the number of leukocytes (S5E Fig). While CD4 cells showed much higher numbers in the infected TAP1-null brain leukocytes, CD8 T cells, γ/δ T cells and NK cells also showed marginal increase (S5E Fig). Phagocytic cell numbers (Gr-1+) were not different between infected and uninfected TAP1-null mice. Neutralizing antibodies could also be detected and were higher in TAP1-null mice as compared to those seen in WT B6 mice (S4B Fig and Fig 6G). In contrast to infected TCRβ-null mice TAP1-null mice did not show breach in the BBB (Relative values for dye-extravasation for infected vs. uninfected TAP1-nulls 1.07 +/- 0.03 vs 1.00 +/- 0.07 from 4–6 mice per group) on day 12 post-infection. Thus, in infected TAP1-null mice there is late onset weight-loss and neurological symptoms as compared to infected TCRβ-null mice correlating with late onset mortality. This is associated with no breach in BBB and poor leukocytic infiltrate on day 12 post-infection. We next examined the role of CD8 cells in the adoptive transfer model. TCRβ-null mice receiving spleen cells from TAP1-null mice containing ~5x10^6^ naïve T cells, majority of them CD4 (S1E Fig), showed poor survival as compared to those mice receiving T cells from WT B6 mice as spleen cell transfer (Fig 6H) and the difference in survival was significant. These data clearly demonstrate that adoptively transferred naïve CD8 T cells from WT B6 mice responded to JEV infection and contributed to better survival of mice. As CD8 T cell mediated IFNγ was not seen to be crucial in contributing to protective immune response to JEV, we next focused on the other major effector contribution of CD8 T cells which is granule mediated cytotoxicity and killing of target cells.

**Fig 7:**
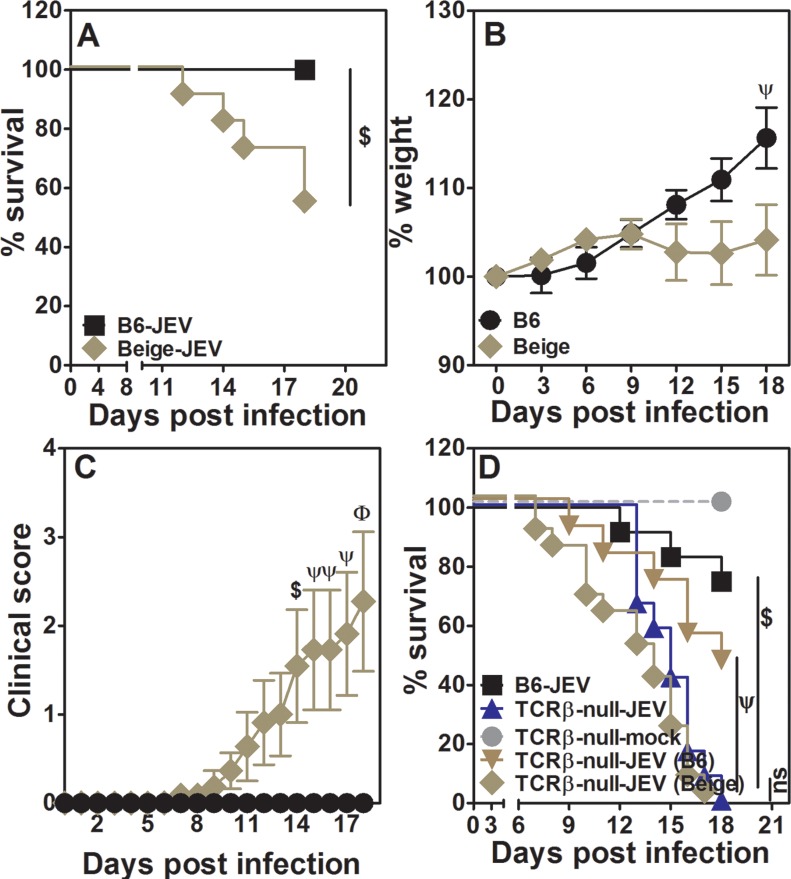
Beige defect makes mice susceptible to JEV infection. [A] Survival kinetics following JEV infection in WT B6 and beige mice over time (n > 8). [B] Relative weight loss of JEV infected beige mice as compared to infected WT B6 mice (mean ± SE, n = 14). [C] Clinical score for JEV infected WT B6 and beige mice (mean ± SE, n = 14). [D] Survival kinetics of mock or JEV infected TCRβ-null mice with or without transfer of naïve T cells from beige or WT B6 mice (n > 8). $ = p<0.05, ψ = p<0.01, φ = p<0.001, ns = not significant.

Beige mice have the LYST mutation and show major defects in granule mediated lysis, primarily in NK cells and CD8 T cells [40,41]. WT B6 and beige mice were infected with JEV and post-infection mortality was scored. Beige mice showed significantly higher mortality than WT B6 mice (Fig 7A). While there was no active weight loss in infected beige mice, the difference in the weights of infected WT B6 and beige mice was significant late in the infection (Fig 7B). This could be explained by increasing morbidity, measured as clinical score, in beige mice with infection (Fig 7C). Beige mice do not have any reported defect in CD4 T cells or B cells which is reflected in high titers of neutralizing antibodies detected on day 12 post-infection (Fig 6G). However, on day 12 post-infection about 60% of infected beige mice show presence of virus in the brain (Fig 6F). Thus, enhanced mortality in beige mice could be due to defective target cell lysis functions of NK as well as CD8 T cells. TCRβ-null mice have normal NK function but lack CD8 cell mediated cytotoxic function and hence we attempted to identify which cell type contributes to protection from JEV infection by transferring spleen cells containing 5x10^6^ total naïve T cells from beige mice to TCRβ-null mice and infecting the recipients 24 h later. These recipient mice survived poorly post infection as compared to those receiving cells from spleens of WT B6 mice (Fig 7D). Interestingly, TCRβ-null mice receiving beige T cells started succumbing to infection earlier than those which received WT B6 cells and were as susceptible as TCRβ-null mice without any cell transfer. These data clearly highlight the role for granule-mediated target cell lysis by CD8 T cells in protection from mortality following JEV infection.

## Discussion

JEV infection is a leading cause of morbidity and mortality in Asia despite a significant proportion of infected individuals not progressing to clinical illness. While a vaccine which can trigger a protective immune response in human host will be immensely useful, which components of adaptive immune response are essential to offer protection in majority of the individuals is still not clear. We undertook these studies to understand the relative importance of T cells in the protective immune response and show that cytolytic function of CD8 T cells is very critical, whereas γ/δ-TCR expressing cells also contribute to protection albeit less efficiently. Our data also suggest a significant role for T cells in preventing breach in BBB leading to clinical manifestations of encephalitis.

Some of the earlier work demonstrating a clear role for T cells in the mouse models of JEV has used 14 day old mice [21,39] as opposed to the adult mice used in our study. Unlike in newborn humans, newborn rodents have poorly developed secondary lymphoid organs and near absence of T and B cells in the periphery [42]. By about one month post birth the immune system in mice resembles that seen in adults [42–44] which makes our animal model more representative of the human situation with respect to JEV immune response than some of the earlier ones. Unlike Nakayama strain used by Larena et.al. [15] or P3 strain used by Li et. al. [45] infection with P20778 strain in B6 lead to lesser mortality in our study. These differences could be attributed to the differences in the mode of inoculation (s.c./i.c. vs i.v.) or the source of the virus (Vero cell-derived vs mouse-brain derived) or the virulence of the strain used which has been reported in other studies with flaviviruses in mouse models [46,47].

Our data on Rag1-null mice confirm the importance of the adaptive immune system in the protection from JEV as reported earlier (reviewed in [48]). For the first time we show that γ/δ TCR expressing T cells contribute significantly to the protection from JEV mortality. γ/δ T cells are known to locate themselves preferentially near epithelial barriers [49]. In animal model of neurocysticercosis with *Mesocestoides corti* infection γ/δ T cells are the main population infiltrating the central nervous system [50]. It has been reported that IFNγ secreted by γ/δT cells in the initial stages is responsible for restricting WNV infection [51]. Despite the morbidity observed in TCRδ-null mice, we cannot detect virus in the brains of these mice (Fig 6F), unlike in TCRβ-null mice, suggesting further complexity in the cellular-molecular basis of evolution of various clinical manifestations. In another report, Vγ1 expressing cells which produced IFNγ were found to be protective whereas Vγ4 expressing cells contributed to the pathology observed following WNV challenge [52]. While we have not further dissected the role of different Vγ families for their relative contribution in the pathogenesis, our data on IFNγ-null mice suggest that IFNγ secreted neither by γ/δ T cells nor by any other cell type makes any observable difference in our model system of JEV infection.

Studies in humans as well as in mice have shown that there is an increase in the levels of proinflammatory cytokines in the cerebro-spinal fluid of non-survivors as compared to the survivors [36,53]. In addition to high viral load, we observed higher levels of IL-6, IL-1β and TNFα mRNA in the brains of TCRβ-null mice as compared to the WT B6 mice which are resistant to disease, this despite the fact that brains from infected WT B6 mice had nearly 30 fold higher number of infiltrating leukocytes including T cells and phagocytic cells. It is possible that the high levels of TNFα secreted locally might be involved in the BBB breach. Studies have shown that TNFα can act as a neuroprotector as well as neurodegenerator during flaviviral infections [54,55]. That TNFα may be one of the key regulators of inflammation leading to BBB breach is supported by the observations that treatment with TNFα inhibitor etanercept prevents the breach as well as CNS inflammation in JEV infected mice [33,53].

We also observe presence of large numbers of CD8 T cells in infected WT B6 mice on day 12 post-infection when these mice neither show presence of the virus, nor clinical symptoms of morbidity, nor a breach in BBB. It can be argued that in the early stages of infection in WT B6 mice, despite low levels of virus in the brain, peripherally primed T cells, more CD8 than CD4, migrate and help in containing viral replication. In their absence, as in infected TCRβ-null mice, viral load goes up by many logs which possibly leads to a breach in the BBB. High viral load may trigger local production of TNFα and that may be responsible for a breach in BBB in TCRβ-null mice which takes place in the near absence of infiltrating T cells and phagocytes. Further, our observations of minimal damage to the BBB in both TAP1-null and MHCII-null mice day 12 post-infection seem to suggest that the presence of α/β TCR bearing T cells may indeed be providing protection from a breach in BBB, either via help provided by CD4 T cells to produce high titer neutralizing antibodies, or via CD8 T cells to lyse infected target cells, or both.

Our finding that IFNγ is not significantly higher in JEV infected brain is consistent with our data on IFNγ-null mice, which show the same level of resistance to JEV infection as WT B6 mice. Using P3 strain of virus it has been shown that IFNγ levels in the brain go up from day 3 onwards [56]. While the same report also shows high levels of IL-6, neither IL-1β nor TNFα was estimated. Using Nakayama strain of JEV it has been shown that IFNγ-null mice are more susceptible to infection than WT B6 mice, unlike our observations and this may be due to differences in the virulence of the strains used [24]. Using an infant mouse model it has been shown that mice surviving JEV infection show higher levels of mRNA for anti-inflammatory cytokines IL-10 and IL-4 [41,57]. We could not detect IL-4 and IL-10 mRNA in the mice which were resistant to the disease. In a mouse model of WNV infection, IL-10-null mice are reported to be more resistant to the infection as compared to WT mice [37]. We do not find any significant role for IL-10 in JEV infection. These data are in direct contrast to the observations reported on WNV infection reiterating the point that pathogenetic mechanisms operative during WNV and JEV infections may be different.

Our data on neutralizing antibody levels suggest interesting possibilities. While WT B6, TCRδ-null, TAP1-null and beige mice show robust generation of neutralizing antibodies, TCRβ-null mice have relatively low levels, despite having high and sustained levels of IgM antibodies. As expected, in the absence of T cell help JEV-specific IgG antibodies are not generated in these mice indicating that neutralizing antibodies seen in TCRβ-null mice be dominantly IgM type. While titers of neutralizing antibodies in TCRβ-null mice are only about 2 fold lower, these antibodies in the absence of α/β TCR bearing T cells are not able to protect. Our data also clearly show that CD8 T cells do not have any role in triggering the neutralizing antibody response since TAP1-null mice, with normal CD4 T cells, have high levels of neutralizing antibodies. Despite robust development of neutralizing antibodies infection in TAP1-null mice and beige mice is associated with relatively high mortality. These data underscore the importance of functional CD8 T cell responses contributing to protection during the course of at least primary infection. During post-primary infections the presence of neutralizing antibodies can change the pathogenesis and outcome of the infection.

Our data on TCRβ-null mice very clearly document the essential role for T cells in providing protection. When we transfer purified naive T cells into TCRβ-null mice, JEV-mediated morbidity is abrogated, indicating that CD8 and/or CD4 T cells are responsible for this protection. We also observe that TAP1-null mice show greater JEV morbidity than MHCII-null mice do, suggesting a greater role for CD8 than for CD4 T cells. This is substantiated by the data where transferring MHCII-null spleen cells, but not TAP1-null spleen cells, abrogates JEV morbidity in TCRβ-null mice. Together, we argue that our data implicate CD8 T cells as crucial components of protection against JEV morbidity in mice.

It has been reported that antiviral antibodies need help from CD4 T cells for reaching neuronal tissues [58]. During first week of infection we and others [56] observe presence of the virus in high titers in the brain. We do not observe JEV-specific IgG antibodies by day 5 post-infection in B6 mice. Thus, high affinity IgG antibodies may not develop enough during the first week of infection and may not reach CNS to have their positive effect. During this window of primary infection CD8 T cells are likely to have a significant impact, especially with localized target killing potential. It has been observed that both cytolytic function as well as IFNγ help in protection of mice when infected with Nakayama strain of JEV which is lethal for WT B6 mice [24]. In the absence of any role played by IFNγ production in protection in our model system, we still observe that cytotoxic function of CD8 T cells is a critical contributor to protection. Our data using beige mice brings about an additional dimension to the parameters of protective immunity required for JEV infection. Beige mice have a defect in endo-lysosomal fusion process and granular exocytosis which adversely affects functioning of both CD8 T cells and NK cells. Beige mice succumb to JEV infection. However, the adoptive transfer approach we have used identifies a major role for CD8 T cells, and not NK cells, in target cell killing to confer protection from JEV associated mortality. Our data confirm earlier report which also showed that NK cells are dispensable during recovery from lethal JEV infection [24]. In experimental WNV infection and vaccine mediated protection studies too CD8 T cells are identified as critical for protection [59–62].

Using adult mouse models lacking various components of immune responses we thus identify importance of various T cell subsets in protective immune response to JEV. For the first time we report that γ/δ T cells do have a significant role to play in protection. We also identify granule-mediated cytotoxic function of CD8 T cells as the critical component of the immune response. These data thus are likely to be useful in designing vaccines where potent cytotoxic T cell response generation can be the important parameter of efficacy.

## Supporting information

S1 Fig Phenotypic characterization of major cell types in the spleens of various strains. [A] Representative staining profiles for B cells, macrophages (myeloid lineage cells), NK cells and γ/δ T cells from WT B6 mouse spleen. [B] Staining for CD4 and CD8 subsets from WT B6 spleen (left), and staining for naïve CD4 (bottom right) and naïve CD8 (top right) T cell subset. [C] Representative staining profile to show CD3+NK1.1+ NKT cells in WT B6 spleen. [D] Pooled data from various mouse strains to show the frequencies of the major cell subsets as indicated (mean + s.e. from 3 mice). [E] Further representation of T cells and their subsets to show frequencies in different mouse strains (mean ± s.e. from 3 mice).
(TIF)

S2 Fig Phenotypic characterization of leukocyte infiltrate in brain and identifying JEV-specific T cells in infected spleen. [A] Representative staining profile of total leukocyte population from infected WT B6 brain identified as CD45+ cells. [B] Gating strategy to identify NK (NK1.1+) cells and phagocytic cells (Gr-1+) from total CD45+ leukocyte population. All CD11b+ve cells were also Gr-1+ve and hence not identified separately. [C] Representative staining profile for CD3+ve cells in total CD45+ leukocytes. [D] Gating strategy for TCRγ/δ +ve and–ve CD3+ve cells from [C]. [E] Staining profile of CD4 and CD8 cells on CD3+TCRγ/δ-ve cells from [D]. [F] Representative figure to show staining of CD44highCD69+ population as activated memory cells in CD4 and CD8 subsets in brain. [G] Representative staining pattern of splenic cells from infected WT B6 mice cultured for 12 h in vitro in presence of JEV to identify CD4 and CD8 T cell populations. [H] Representative figure to show staining of CD44highCD69+ memory cell frequencies in response to JEV in CD4 and CD8 subsets.
(TIF)

S3 Fig Viral titers in various organs. Viral titers by qRT-PCR in various organs of infected WT B6 and TCRβ-null mice 2 (top) and 4 (bottom) days post infection. Each symbol represents data from one mouse.
(TIF)

S4 Fig Plaque assays for determining neutralizing antibody titers. [A] Representative images showing plaques for serum at various dilutions, as indicated against each well, from control, uninfected WT B6 mouse (left) and infected WT B6 mouse (right). [B] Images as in [A] for serum from control, uninfected (left) and infected (right) TAP1-null mouse each. [C] Images for serum from control, uninfected (left) and infected (right) TCRβ-null mouse each. Images from TCRδ-null and beige mouse sera not shown.
(TIF)

S5 Fig Effect of absence of IL-10 or IL-4 on JEV infection and phenotyping leukocytes from TAP1-null mice. [A] Survival kinetics following JEV infection in WT B6 and IL-10-null mice over time (n > 8). [B] Survival kinetics of mock or JEV infected TCRβ-null mice with or without transfer of naïve T cells from IL-10-null or WT B6 mice (n > 8). [C] Survival kinetics following JEV infection in WT B6 and IL-4-null mice over time (n > 8). [D] Survival kinetics of mock or JEV infected TCRβ-null mice with or without transfer of naïve T cells from IL-4-null or WT B6 mice (n > 8). [E] Distribution of leukocyte subsets per brain in uninfected WT B6, uninfected TAP1-null and infected TAP1-null mice (mean + SE, n as shown). $ = p<0.05, ψ = p<0.01, ns = not significant.
(TIF)
