# Supplementary figures and images for: CD8 T cells protect adult naive mice from JEV-induced morbidity via lytic function
Source: PLoS Negl Trop Dis. 2017 Feb 2;11(2):e0005329. doi: 10.1371/journal.pntd.0005329 (PMC5308832; doi:10.1371/journal.pntd.0005329)

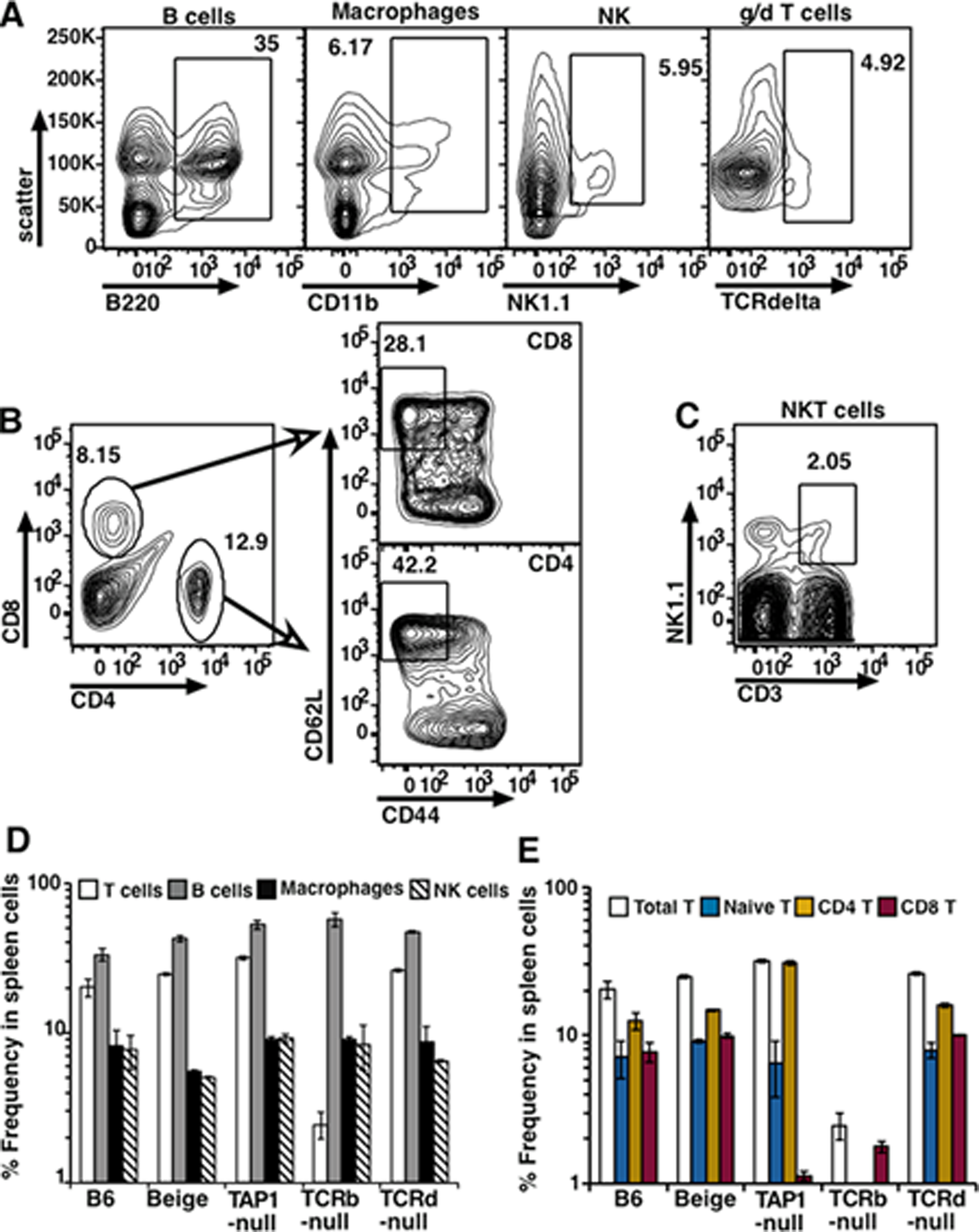

Supplement: S1 Fig — [A] Representative staining profiles for B cells, macrophages (myeloid lineage cells), NK cells and γ/δ T cells from WT B6 mouse spleen. [B] Staining for CD4 and CD8 subsets from WT B6 spleen (left), and staining for naïve CD4 (bottom right) and naïve CD8 (top right) T cell subset. [C] Representative staining profile to show CD3+NK1.1+ NKT cells in WT B6 spleen. [D] Pooled data from various mouse strains to show the frequencies of the major cell subsets as indicated (mean + s.e. from 3 mice). [E] Further representation of T cells and their subsets to show frequencies in different mouse strains (mean ± s.e. from 3 mice). (TIF) [file pntd.0005329.s001.tif]

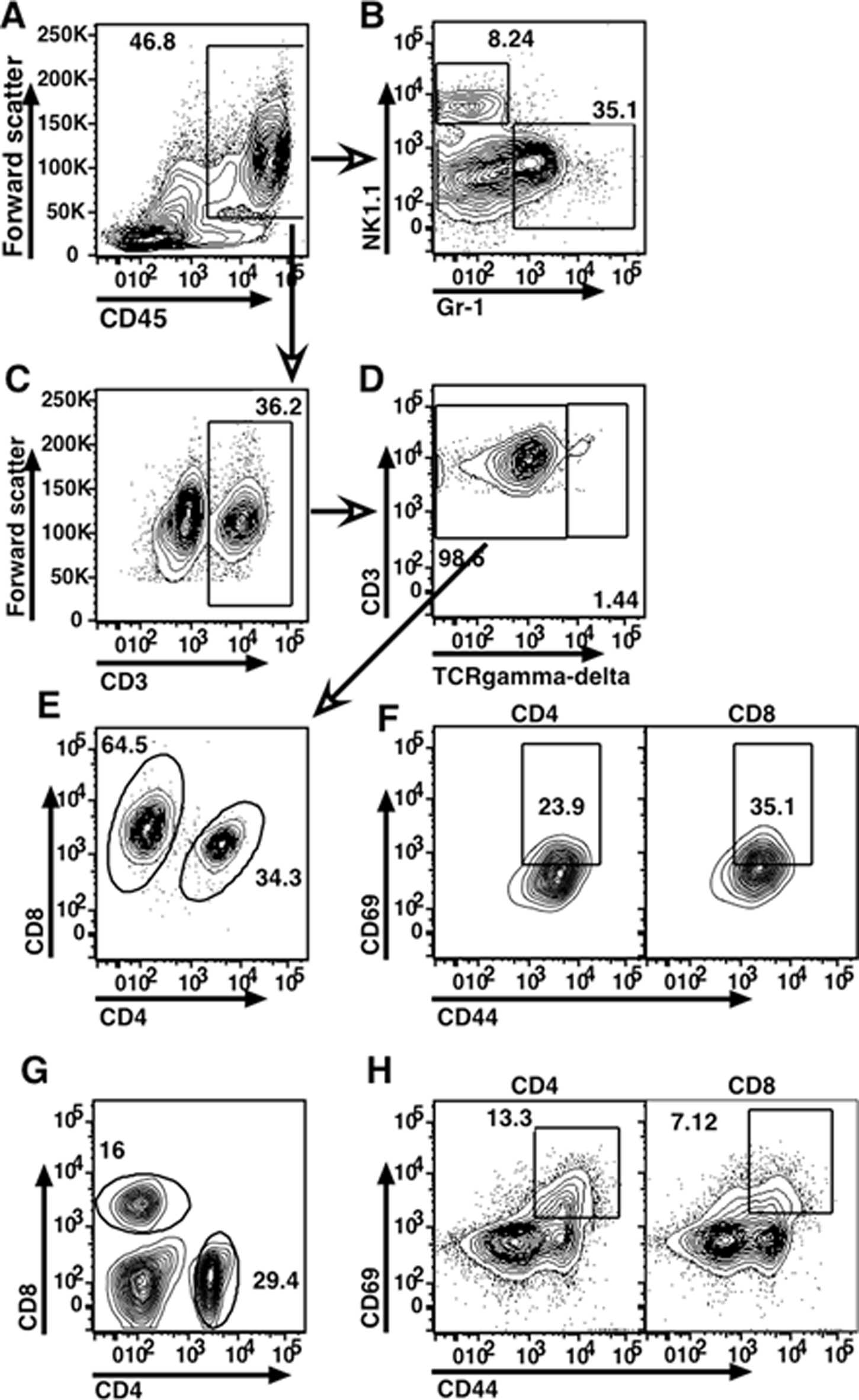

Supplement: S2 Fig — [A] Representative staining profile of total leukocyte population from infected WT B6 brain identified as CD45+ cells. [B] Gating strategy to identify NK (NK1.1+) cells and phagocytic cells (Gr-1+) from total CD45+ leukocyte population. All CD11b+ve cells were also Gr-1+ve and hence not identified separately. [C] Representative staining profile for CD3+ve cells in total CD45+ leukocytes. [D] Gating strategy for TCRγ/δ +ve and–ve CD3+ve cells from [C]. [E] Staining profile of CD4 and CD8 cells on CD3+TCRγ/δ-ve cells from [D]. [F] Representative figure to show staining of CD44highCD69+ population as activated memory cells in CD4 and CD8 subsets in brain. [G] Representative staining pattern of splenic cells from infected WT B6 mice cultured for 12 h in vitro in presence of JEV to identify CD4 and CD8 T cell populations. [H] Representative figure to show staining of CD44highCD69+ memory cell frequencies in response to JEV in CD4 and CD8 subsets. (TIF) [file pntd.0005329.s002.tif]

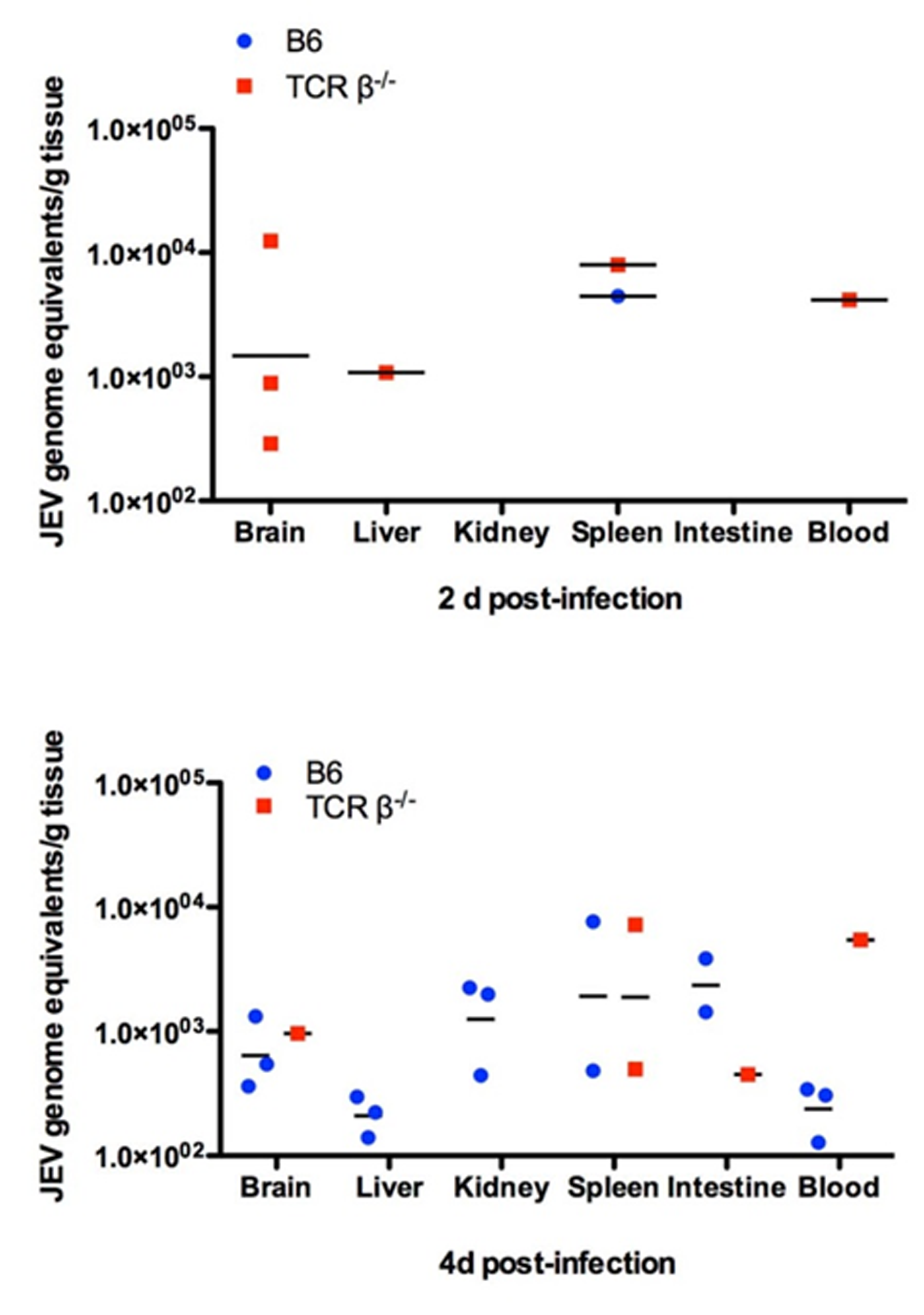

Supplement: S3 Fig — Viral titers by qRT-PCR in various organs of infected WT B6 and TCRβ-null mice 2 (top) and 4 (bottom) days post infection. Each symbol represents data from one mouse. (TIF) [file pntd.0005329.s003.tif]

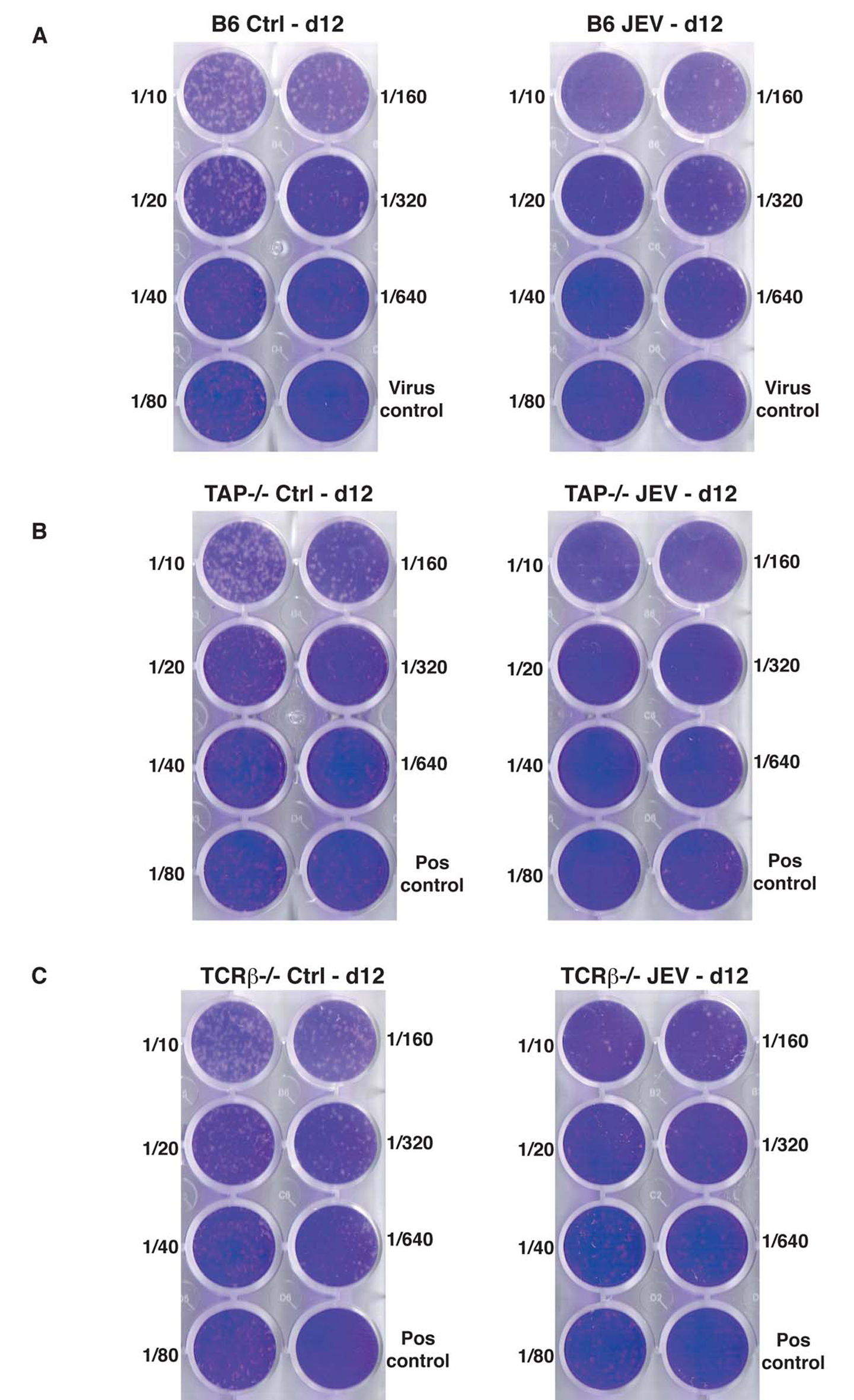

Supplement: S4 Fig — [A] Representative images showing plaques for serum at various dilutions, as indicated against each well, from control, uninfected WT B6 mouse (left) and infected WT B6 mouse (right). [B] Images as in [A] for serum from control, uninfected (left) and infected (right) TAP1-null mouse each. [C] Images for serum from control, uninfected (left) and infected (right) TCRβ-null mouse each. Images from TCRδ-null and beige mouse sera not shown. (TIF) [file pntd.0005329.s004.tif]

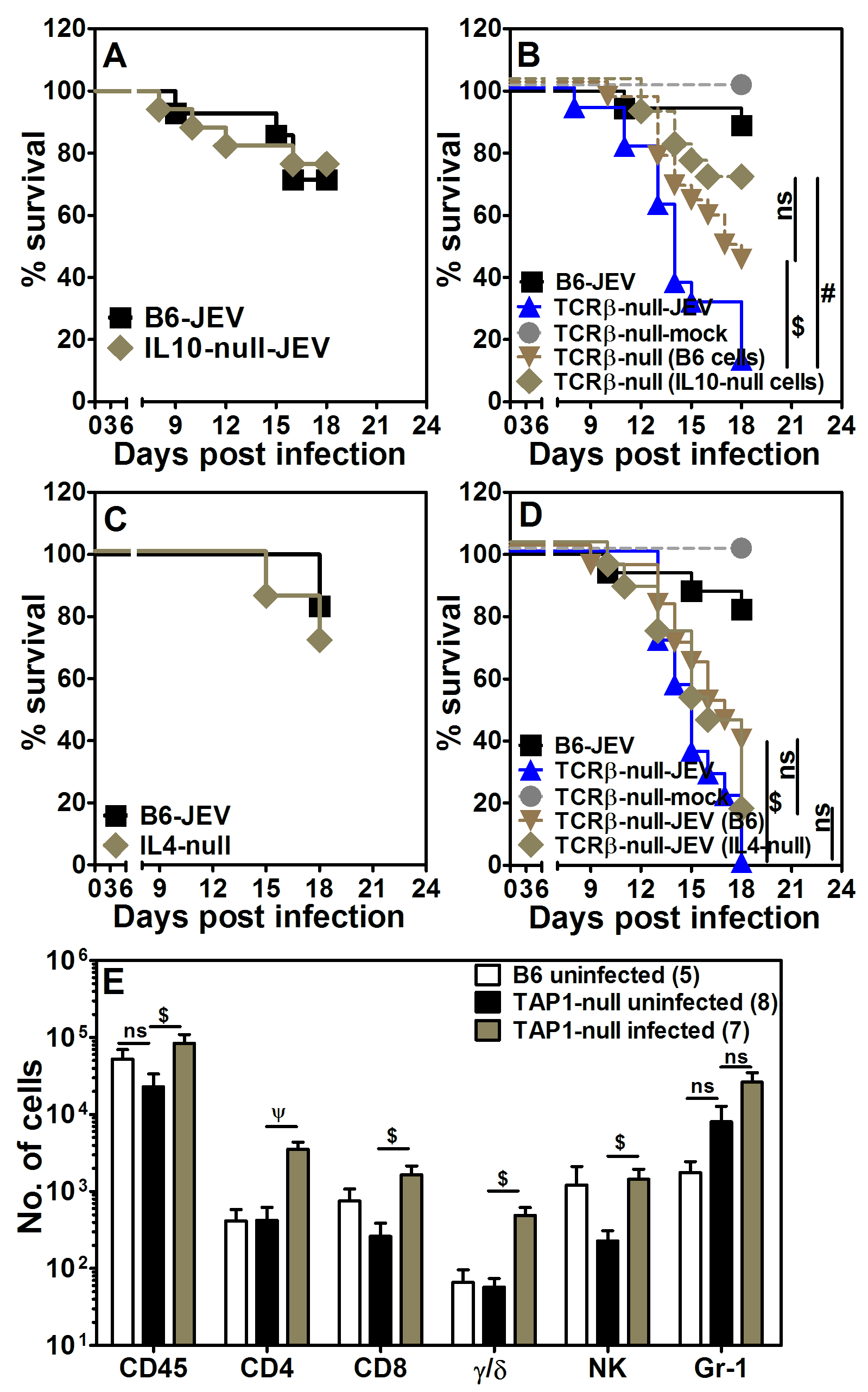

Supplement: S5 Fig — [A] Survival kinetics following JEV infection in WT B6 and IL-10-null mice over time (n > 8). [B] Survival kinetics of mock or JEV infected TCRβ-null mice with or without transfer of naïve T cells from IL-10-null or WT B6 mice (n > 8). [C] Survival kinetics following JEV infection in WT B6 and IL-4-null mice over time (n > 8). [D] Survival kinetics of mock or JEV infected TCRβ-null mice with or without transfer of naïve T cells from IL-4-null or WT B6 mice (n > 8). [E] Distribution of leukocyte subsets per brain in uninfected WT B6, uninfected TAP1-null and infected TAP1-null mice (mean + SE, n as shown). $ = p<0.05, ψ = p<0.01, ns = not significant. (TIF) [file pntd.0005329.s005.tif]
